# Encapsulation Properties of *Mentha piperita* Leaf Extracts Prepared Using an Ultrasound-Assisted Double Emulsion Method

**DOI:** 10.3390/foods12091838

**Published:** 2023-04-28

**Authors:** Bhawna Sobti, Afaf Kamal-Eldin, Sanaa Rasul, Mariam Saeed Khalfan Alnuaimi, Khulood Jaber Jasim Alnuaimi, Alia Ali Khsaif Alhassani, Mariam M. A. Almheiri, Akmal Nazir

**Affiliations:** Department of Food Science, College of Agriculture and Veterinary Medicine, United Arab Emirates University, Al Ain P.O. Box 15551, United Arab Emirates

**Keywords:** peppermint extract, encapsulation, ultrasonic extraction, phenolic content, plant proteins

## Abstract

Double emulsions (W_1_/O/W_2_) have long been used in the food and pharmaceutical industries to encapsulate hydrophobic and hydrophilic drugs and bioactive compounds. This study investigated the effect of different types of emulsifiers (plant- vs. animal-based proteins) on the encapsulation properties of *Mentha piperita* leaf extract (MLE) prepared using the double emulsion method. Using response surface methodology, the effect of ultrasound-assisted extraction conditions (amplitude 20–50%; time 10–30 min; ethanol concentration 70–90%) on the total phenolic content (TPC) and antioxidant activity (percent inhibition) of the MLE was studied. MLE under optimized conditions (ethanol concentration 76%; amplitude 39%; time 30 min) had a TPC of 62.83 mg GA equivalents/g and an antioxidant activity of 23.49%. The optimized MLE was encapsulated using soy, pea, and whey protein isolates in two emulsifying conditions: 4065× *g*/min and 4065× *g*/30 s. The droplet size, optical images, rheology, and encapsulation efficiency (EE%) of the different encapsulated MLEs were compared. The W_1_/O/W_2_ produced at 4065× *g*/min exhibited a smaller droplet size and higher EE% and viscosity than that prepared at 4065× *g*/30 s. The higher EE% of soy and pea protein isolates indicated their potential as an effective alternative for bioactive compound encapsulation.

## 1. Introduction

*Mentha piperita*, the peppermint (mint) plant, is a well-known aromatic herb belonging to the *Lamiaceae* family. It was first cultivated in the Mediterranean basin and is indigenous to Europe and the Middle East [1]. *M. piperita* has a characteristic sharp smell and flavor; thus, it is extensively used as a flavoring in foods and for culinary purposes. Herbal tea, chewing gums, and mint capsules are other popular mint-flavored products available in the market. Peppermint leaves have many health and therapeutic benefits, such as antioxidant, antitumor, antimicrobial, antiallergenic, and immunomodulating actions, as well as gastrointestinal benefits [2,3]. Moreover, in vitro studies have demonstrated the potential antidepressant effects of mint extracts [4]. Most of these physiological and therapeutic effects are associated with the high phenolic content of peppermint leaves (approximately 19–23%) [5,6]. Peppermint also exhibits a high radical scavenging activity and anti-HIV properties because of the presence of high amounts of flavonoids, especially luteolin 7-*O*-b-glucuronide [7].

Despite the much-appreciated benefits of phenolic compounds for human health, supplementation and inclusion directly in the diet is limited and difficult. Thus, the extraction of these compounds from the leaves of peppermint plants is the best way to utilize them in different food and medicinal applications, such as in the form of a *Mentha piperita* leaf extract (MLE). These phenolic compounds have been traditionally extracted from the plant matrix using the Soxhlet method; however, this technique has many disadvantages, such as a long processing time (several hours to days), the requirement for large amounts of solvent, high energy consumption, high rejection of CO_2_, and potential degradation of bioactive compounds [8]. These shortcomings of the conventional extraction method have forced the food industries to use “green techniques,” such as ultrasonic extraction (UE), which are associated with low energy costs and lower solvent consumption. Ultrasound can be used in the phytopharmaceutical extraction industry as a potential technology for the preparation of a wide range of herbal extracts [9]. Ultrasound waves enable an efficient and superior extraction of bioactive compounds via their cavitational effects, which accelerate heat and mass transfer, thus speeding up the release of these compounds by disrupting the plant cell walls. Furthermore, UE can be performed at a relatively low temperature, which protects heat-sensitive compounds from degradation or volatilization. Many studies have reported the UE of bioactive principles from herbs; however, the research data that have been collected in this context for peppermint leaves are scarce [10,11,12,13].

The bioactive agents present in MLEs cannot be used directly because they are poorly water-soluble, chemically unstable, and highly susceptible to degradation at high processing conditions or during storage (due to their sensitivity to oxygen and light); moreover, they may have limited biological activity, which restricts their applicability and compromises their valuable effects on human health [14,15,16]. Consequently, encapsulation of MLEs may improve not only their bioavailability and controlled release [17,18] but also their stability, and may mask their astringency and bitterness [19]. Among the encapsulation techniques available, double emulsion is a promising concept because of its unique advantage of encapsulating both the lipophilic and hydrophilic bioactive compounds that are generally found in plant extracts [20,21,22,23]. In this study, a W_1_/O/W_2_ (water-in-oil-in water)-type of double emulsion was used for encapsulation, where water droplets (W_1_) were dispersed in oil globules which were subsequently dispersed in a continuous aqueous phase (W_2_).

Double emulsions are considered thermodynamically unstable, as they undergo creaming, separation, flocculation, and coalescence [24,25]. Several articles in the literature reviewed the effect of the particle size of both the water droplets and oil droplets on the stability of the resulting double emulsions [26]. Other optimization attempts, such as modulating the volume fractions and viscosity of the two phases and emulsifier concentrations, monitoring the processing conditions of emulsification (speed, duration, and method), and steric stabilization [27,28,29,30] have provided improvements in the stability of double emulsions.

In turn, the addition of macromolecules (such as proteins) to the internal and/or external phases afforded a considerable improvement in the stability of multiple emulsions [31,32] via steric stabilization and electrostatic repulsion effects. In recent years, the use of plant proteins has attracted tremendous interest for the encapsulation of drugs and bioactive compounds because of its low cost, high nutritional value, good emulsifying properties, and lower allergenicity compared with animal proteins [33,34]. In this study, soy protein isolates (SPI) and pea protein isolates (PPI) were used as an encapsulating material for the production of W_1_/O/W_2_ double emulsions to evaluate their encapsulation efficiency (EE) compared with whey protein isolates (WPI). Many studies have reported the use of dairy proteins as a delivery vehicle for bioactive compounds [35,36,37]. In contrast, the work published on the application of plant proteins as encapsulating agents, such as α-tocopherols [38], tomato oleoresins [39], etc., is limited and no detailed study has been conducted to date to examine their effects on MLE encapsulation.

Based on the information provided above, the objectives of our study were to (1) optimize the conditions (amplitude, time, and ethanol concentration) of the ultrasound-assisted extraction of phenolic compounds from peppermint leaves and (2) study the encapsulation properties of the MLEs within W_1_/O/W_2_ emulsions prepared using different coating materials (plant-based proteins vs. animal proteins). This study provided a clear understanding of the types of coating material that afford better emulsion stability; thus, it can be expected to have high applicability to the relevant industries.

## 2. Materials and Methods

*Mentha piperita* leaves were collected from the local supermarket of Alain, UAE. Commercial food-grade PPI (80% protein) and SPI (90% protein) were purchased from MYVEGAN^TM^. Sunflower oil and WPI (92% protein, Gold Standard, Downers Grove, IL, USA) were obtained from the local supermarket of Alain, UAE. Chemicals, viz. Folin–Ciocalteu and 2, 2-diphenyl-1-picrylhydrazyl (DPPH), were purchased from Sigma Chemical Corp. (St. Louis, MO, USA).

### 2.1. Peppermint Leaf Powder Preparation

Fresh peppermint leaves were washed under water to remove any dirt and dust and then dried in an oven at 60 °C for 24 h until the moisture content was constant (8.5%). The dried mint leaves were powdered using a 50-mesh sieve, vacuum-packed in a plastic bag, and kept in a freezer at −20 °C for further use.

### 2.2. Ultrasonic-Assisted Extraction

Peppermint leaf extraction was performed using a probe sonicator (Branson Sonifier^®^, Danbury, CT, USA) operating at a frequency of 40 kHz with an input power of 150 W and a sonotrode with a diameter of 6 mm. Briefly, ultrasonic-assisted extraction of phenolic compounds was performed under the following conditions: ethanol conditions 70–90%, amplitude 20–50% and time 10–30 min. For each experiment, the dried mint powder: solvent ratio was 1:30 (*w*/*w*) [40,41]. During the extraction process, the temperature of the sample was controlled by employing an ice bath. After extraction, the samples were centrifuged at 4065× *g* for 15 min at 20 °C and the supernatants were filtered using Whatman filter paper No.1 in a 50-mL volumetric flask, with the final volume made up to 50 mL using solvent. The MLE was then concentrated in a rotary evaporator at 60 °C under vacuum conditions. The final MLE was stored in a refrigerator at 4 °C before further analysis.

#### Experimental Design for Optimization of UE of Peppermint Leaves

A response surface methodology (RSM) with a Box–Behnken design was employed to study the effect of independent variables (amplitude, time, and ethanol concentration) at three levels (–1, 0, +1) on the two outcome parameters (total phenolic content (TPC) and antioxidant activity). A total of 15 experiments (including 3 center points) were performed to optimize the operating conditions, consisting of amplitude (X_1_) (20–50%), time (X_2_) (10–30 min), and ethanol concentration (X_3_) (70–90%), as shown in Table 1. A quadratic polynomial model was fitted to each outcome using the following equation:

(1) $$y=\beta_{0}+\sum_{i=1}^{4}\beta_{i}x_{i}+\sum_{i=1}^{4}\beta_{ii}x_{i}^{2}+\sum_{i=1}^{3}\sum_{j=i+1}^{4}\beta_{ij}x_{i}x_{j}$$

where y is the response, β_0_ is a constant, β_i_ is the linear coefficient, β_ii_ is the quadratic coefficient, β_ij_ is the interaction coefficient, and X_i_ and X_j_ are independent variables. Next, the TPC and antioxidant activity were measured at each experimental run. The optimization was carried out based on the optimization settings presented in Table 2. Using the optimized factor conditions, specific outcomes were predicted and validated.

### 2.3. Analysis of Extract

#### 2.3.1. Determination of TPC

The total phenolic compound content of the MLE was determined using the Folin–Ciocalteu method, as described previously by Jabri-Karoui et al. [42]. Briefly, a 60-µL aliquot of the MLE was mixed with 1.5 mL of diluted Folin–Ciocalteu reagent (2 M). The resulting solution was left for 5 min at room temperature (15 °C) and then mixed with 2 mL of saturated Na_2_CO_3_ solution. Next, the total volume was made up to 4.5 mL with water, followed by thorough vortexing. The mixture was incubated at room temperature (20 °C) in a dark place for 30 min, and the absorbance of each sample was read at a wavelength of 760 nm against a blank with no extract addition. Each analysis was performed in triplicate. The TPC of the MLE was expressed as mg gallic acid equivalents (GAE)/g of dry weight using the gallic acid standard curve equation (y = 0.0081x + 0.0321, *R*^2^ = 0.99).

#### 2.3.2. Quantitative Analysis of Antioxidant Activity Using DPPH Method

The antioxidant activity in terms of percent inhibition for MLE were determined using DPPH stable free radical assay as described by Sadef et al. [43]. The DPPH solution was prepared by dissolving 7 mg of DPPH in 55 mL of methanol (0.3 mM DPPH). In a test tube, 20 µL of test sample was added to 2 mL of DPPH solution. The final volume was adjusted to 4 mL using methanol, followed by vortexing. The mixture was kept undisturbed in the dark for 30 min at ambient temperature (20 °C). Control samples containing the same amount of methanol and DPPH solution were also prepared. The absorbance was measured spectrophotometrically at 515 nm. The percentage inhibition was then calculated using the following formula:

% inhibition = Absorbance (control) − Absorbance (sample)/Absorbance (control) × 100(2)

### 2.4. Encapsulation of MLE Using Double Emulsion Method

Emulsions were prepared according to the method described by Aditya et al. [44] with minor modifications. The preparation of a double emulsion involves two steps: (1) preparation of the inner water-in-oil (W_1_/O) emulsion, where W_1_ droplets are dispersed in the oil phase; (2) preparation of a water-in-oil-in-water (W_1_/O/W_2_) emulsion system, in which the primary emulsion (W_1_/O) is dispersed in an outer water phase (W_2_).

#### 2.4.1. Preparation of Inner W_1_/O Emulsion

The inner aqueous phase (W_1_) was prepared by first dissolving the MLE in a pure NaCl solution (0.1 M). Subsequently, the W_1_/O emulsion was prepared by dispersing the inner aqueous phase containing MLE (20%, *w*/*w*) in a mixture of sunflower oil (60%, *w*/*w*) and Span 80 (0.2%, *w*/*w*) using an ULTRA-TURRAX homogenizer (T25 D, IKA^®^-Werke GmbH & Co. KG, Staufen, Germany) at 4065× *g* for 2 min.

#### 2.4.2. Preparation of W_1_/O/W_2_ Emulsion

Double emulsions were prepared using 25% (*w*/*w*) of W_1_/O and 75% (*w*/*w*) of an outer aqueous phase (W_2_). The outer aqueous phase consisted of a 10% protein solution (soy protein isolates (SPI), pea protein isolates (PPI), or whey protein isolates (WPI)) or a control solution (0.5% Tween 80). The protein solutions were prepared by dissolving 10% protein in distilled water followed by mixing via magnetic stirring for 2 h. In addition, sodium azide (0.02%, *w*/*v*) was added as a microbial inhibitor. The solutions were kept in a refrigerator overnight at 4 °C. Subsequently, the protein solutions were decanted and centrifuged at 4065× *g* for 15 min at 4 °C, to obtain a clear supernatant, which was then used as the outer aqueous phase (W_2_). For W_1_/O/W_2_ preparation, the primary emulsion was gradually added to the W_2_ phase and was homogenized using an ULTRA-TURRAX homogenizer (T25 D, IKA^®^-Werke GmbH & Co. KG, Staufen, Germany) at 4065× *g* for 1 min and 30 s. The time of homogenization for encapsulation was optimized by studying different emulsion characteristics.

### 2.5. Characterization of Emulsions

#### 2.5.1. Optical Microscopy and Droplet Size Analysis of Emulsions

The W_1_/O/W_2_ emulsions were examined using an optical microscope (Delphi-X Observer^TM^, Euromex, Arnhem, The Netherlands). One drop of freshly prepared emulsion was placed on a glass microscope slide and was diluted with two drops of 0.5% Tween 80. A cover slip was placed on it without forming any air bubbles and images were captured using the Image Focus Alpha software using optical microscope at a magnification of 20×.

The size of 25 droplets was measured in micrometers (µm) using the radius tool in the Image Focus Alpha software, and was expressed as the mean ± SD.

#### 2.5.2. Viscosity Measurement

The viscosity of emulsions was recorded using a Discovery HR-3 rheometer (TA Instruments, New Castle, DE, USA). All measurements were performed at 25 °C using cylinder geometry at a gap of 300 µm. The apparent shear viscosity was determined from measurements of the shear stress vs. shear rate when the shear rate was increased from 1 to 10 s^−1^ [45].

#### 2.5.3. Encapsulation Efficiency (EE)

The EE % of the W_1_/O/W_2_ emulsions was measured as the TPC that remained in the inner emulsion (W_1_/O) after the second emulsification step, as per Gustavo et al. [46], with minor modifications. Briefly, 1 mL of the double emulsion sample was centrifuged at two speeds, i.e., 15,596× *g* for 10 min at 4 °C, clearly separating the supernatant from the lipid. The nonencapsulated phenolic compounds in the supernatant obtained after centrifugation was assessed according to the Folin–Ciocalteu method using gallic acid as a standard to prepare the calibration curve. Phenol content was expressed as mg equivalents of gallic acid per gram of extract [47]. The percentage of encapsulated compounds was determined using the following formula [48]:

EE% = (Total amount of bioactive compound (mg) − Amount of bioactive compound in supernatant (mg))/Total amount of bioactive compound × 100(3)

### 2.6. Statistical Analysis

The UE experiments were optimized using a Box–Behnken design, and contour plots of the model were obtained using the Minitab^®^ 18 statistical software. The significance of the model was tested using an analysis of variance with *R*^2^ > 0.70 to determine whether the models could be used to predict the response of an outcome parameter to the independent variables. The encapsulation results were subjected to one-way analysis of variance (ANOVA) using SPSS version 25.0 (SPSS, Inc., Chicago, IL, USA). Significant differences (*p* < 0.05) were determined using Tukey’s a,b test.

## 3. Results and Discussion

### 3.1. Optimization of TPC and Percent Inhibition Using RSM

Phenolic compounds are widely known for their radical scavenging activity and are used as reducing agents, hydrogen or electron donors, and metal chelators [49]. Moreover, they exhibit good antioxidant and anti-inflammatory properties, which play important roles in human health. Table 1 shows that the lowest TPC was detected at the extraction condition using an amplitude of 35% for 10 min and an ethanol concentration of 90%, whereas the highest TPC extraction levels occurred by treating the mint leaf powder using an amplitude of 35% for 30 min and an ethanol concentration of 70%. Yi and Wetzstein [50] and Uribe et al. [51] extracted the phenolic compounds of *M. piperita* L. via extraction methods using normal shaking or stirring and reported that longer extraction times afforded lower or similar extraction yields. Furthermore, Uribe et al. [51] reported that extraction using the stirring method using 50% methanol for 2 h yielded the highest TPC (17.81 mg GA equivalents/g) from peppermint leaves (vacuum dried at 60 °C). Similarly, Yi and Wetzstein [50] obtained the highest TPC (38 mg GA equivalents/g) when extracting phenolic compounds from a peppermint powder (oven-dried at 70 °C) via a conventional stirring method using 80% ethanol for 2 h. In the current study, UE yielded a higher TPC, mainly because of the cavitational effects of the high-intensity ultrasound waves. This effect resulted in small bubbles filled with gas or vapor, which have been found to undergo irregular oscillations and finally implode. This further produces high local temperatures and pressures, which leads to the disintegration of biological cells and an increase in mass transfer and subsequently enables the fast extraction of bioactive compounds [52]. According to the results presented in Table 2, the linear effect on the TPC value was highly significant (*p* < 0.001) regarding the ethanol concentration alone, whereas the interactive terms were non-significant (*p* > 0.05). The following regression equation, which indicates the effect of amplitude, time, and ethanol concentration on the TPC (Equation (3)), was obtained for significant terms (*p* < 0.05) according to RSM:

TPC = 58.78 − 11.39 × E − 11.27 × E × E (4)

Table 2 shows that the model *p*-value was significant with a very high degree of fit, as indicated by an *R*^2^ > 0.93 value. Significant components made an impressive contribution to the prediction of the respective model. Moreover, the predicted (65.41 mg GA equivalents/g of powder) and experimental (62.8 mg GA equivalents/g of powder) TPC values were in good agreement. Higher TPC values were detected at the longer extraction time when using an ethanol concentration of 80%. This was probably caused by the fact that longer ultrasound-assisted extraction times yielded higher TPC values. Moreover, the type of solvent and its concentration are critical elements of the extraction, as described by [50,53]. In addition, an ethanol concentration of 80% yielded a significantly (*p* < 0.05) higher TPC extraction from dried mint leaves vs. 80% methanol [48], which agrees with our findings. Another study also confirmed that the use of an 80% aqueous ethanol solution afforded a better extraction of phenolic compounds from plant materials [52]. The extraction yield increases with an increase in polarity. By increasing the polarity of the solvent, the solvent system can extract phenolic substances from both ends of the polarity (high-polarity substances and low-polarity substances) [54].

Because of its various health benefits, peppermint has attracted the attention of many researchers toward the exploration of its antioxidant potential. It is well known that antioxidants can reduce oxidative damage to cells and tissues by detoxifying free radicals and can prevent oxidative-stress-related human illness [55]. The results listed in Table 1 show that the highest percent inhibition (as a measure of antioxidant activity) was obtained by extraction at 35% amplitude for 10 min using 70% ethanol, whereas the lowest percent inhibition was recorded at 20% amplitude for 20 min using 90% ethanol. According to the ANOVA (Table 2), the linear effect of amplitude and ethanol, and the interaction effect of time with amplitude and ethanol of the MLE on percent inhibition, were significant (*p* < 0.05). According to the experimental design, the regression equation (Equation (4)) indicating the effect of amplitude, time, and ethanol concentration on the DPPH values of the MLE samples was as follows:

30.63 + 5.67 × X_a_ − 8.65 × X_e_ − 6.5 × X_a_ × X_t_ + 5.13 × X_t_ × X_e_. (5)

**Figure 1:**
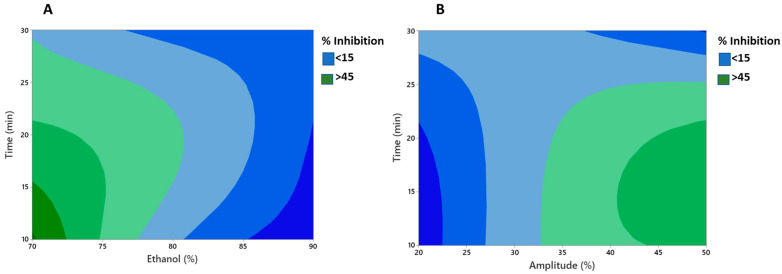
Contour plots showing significant interactive effect between (**A**) time and ethanol (**B**) time and amplitude on % inhibition of UE mint extract.

The lack of fit was insignificant (*p* > 0.05) for antioxidant activity. The high *R*^2^ values (0.86) obtained suggested the high efficiency of the quadratic model for fitting the data under the conditions of the experiment. Moreover, Figure 1A,B demonstrated clearly the interactive effect of different variables, viz. amplitude, time, and ethanol, on the antioxidative activity (percent inhibition) of the MLE. Figure 1A showed that with increase in the ethanol concentration and time of extraction, percent inhibition decreased significantly (*p* < 0.05). It is important that the extraction time be short enough to prevent bioactive components from degrading, resulting in decreased antioxidative activity [56]. In addition, Figure 1B showed that higher amplitude and longer time increased the antioxidant activity of the MLE (*p* < 0.05). It is generally observed that an increase in the amplitude levels strengthens the cavitational bubble effect, because the resonant bubble size is proportional to the amplitude of the ultrasonic wave [57]. However, amplitude levels greater than 35% resulted in a decrease in percent inhibition values, which may be attributed to the degradation of the plant material [58].

For optimization, TPC was taken into consideration, as the model *p*-value was highly significant. The optimum operating conditions for UE extraction were found to be ethanol concentration 76%, amplitude 39% and time 30 min. The TPC and antioxidant property (percent inhibition) at optimized conditions were found to be 62.83 ± 2.35 mg GA equivalent/g mint powder and 23.49 ± 0.63%. In the future, hydrodynamic cavitation could be employed to achieve better extraction yields, higher energy efficiencies, shorter extraction time and easy scalability compared to acoustic-cavitation-based processes [59,60].

### 3.2. Encapsulation of Phenolic Compounds

#### 3.2.1. Optical Imaging and Determination of Droplet Size of Double Emulsions

**Figure 2:**
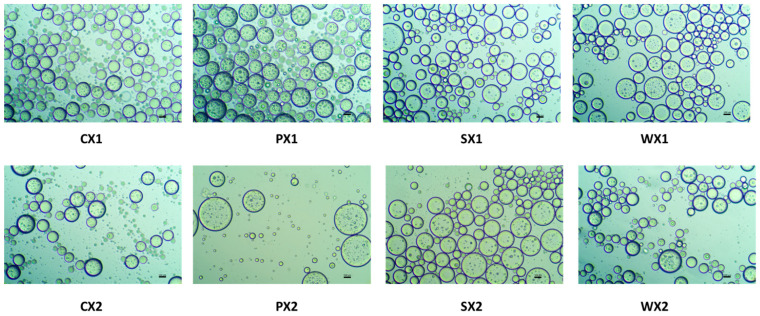
Optical images of double emulsions for two different time durations (C: Control, P: PPI, S: SPI, W: WPI; X1: 4065× *g*/1 min and X2: 4065× *g*/30 s) (×20 magnification; bar = 100 µm).

To obtain a stable double emulsion, a standard two-step emulsification procedure was adopted. A lipophilic emulsifier was used in the first step to prepare the primary W_1_/O emulsion, followed by the addition of a hydrophilic emulsifier in the second step to obtain the W_1_/O/W_2_ emulsion. Moreover, the samples were treated with an electrolyte (e.g., NaCl), which induces a balance between the Laplace and osmotic pressures, thus preventing water migration between the W_1_ phase and the W_2_ phase and improving encapsulation properties [61]. Figure 2 presents optical images of the double emulsions prepared using different protein isolates (PPI, SPI, and WPI) and the control (0.5% Tween 80) at two different emulsification intervals, i.e., 1 min and 30 s. Briefly, all optical micrographs obtained as described above revealed a characteristic structure of the double emulsions, comprising small water droplets within an oil globule. Most of these droplets were multinucleated and had a smooth, round, and well-layered wall, which probably afforded better protection to the core.

**Figure 3:**
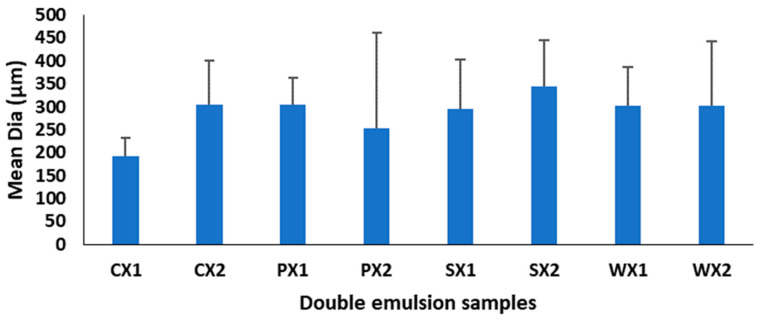
Mean diameter of double emulsions emulsifying at two different times (C: Control, P: PPI, S: SPI, W: WPI; X1: 4065× *g*/1 min and X2: 4065× *g*/30 s; n = 25).

Multiple emulsions commonly undergo breakdown through mechanisms such as gravitational separation, flocculation, and coalescence [62]. Therefore, the effect of different biopolymers on the stability of the emulsions was investigated. Droplet size is a very important criterion for the stability of the emulsification and encapsulation processes. A larger droplet size is related to reduced kinetic stability of the emulsion. In this study, although the mean droplet diameters of the W_1_/O/W_2_ at two emulsification intervals were not significantly different (*p* < 0.05) (Figure 3), samples prepared at 4065× *g* for 30 s exhibited a huge variation in droplet sizes and were heterogeneous. It is possible that the emulsification time (i.e., 30 s) was insufficient to allow a good dispersion of the primary emulsion during the continuous phase, leading to coalescence and aggregation and affecting the stability of the emulsion [63]. Based on size and the uniformity of droplet sizes, the emulsification conditions of 4065× *g* for 1 min yielded better stability of the W_1_/O/W_2_. Moreover, all plant proteins, but especially PPI, had a droplet diameter that was equivalent to that of WPI, which indicates their potential as an alternative to animal proteins for the encapsulation of MLE bioactive compounds.

#### 3.2.2. Viscosity Measurements

**Figure 4:**
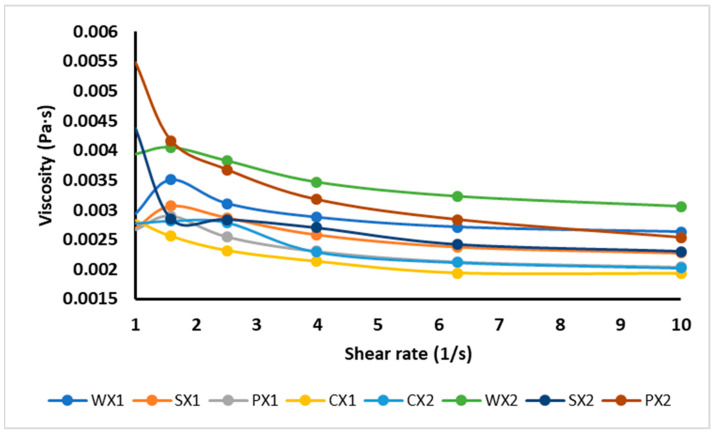
Viscosity of double emulsions emulsifying at two different times (C: Control, P: PPI, S: SPI, W: WPI; X1: 4065× *g*/1 min and X2: 4065× *g*/30 s).

The effect of different types of emulsifiers on viscosity is reported in Figure 4. Viscosity is the main factor determining the physical stability of the W_1_/O/W_2_, as it prevents phase separation. It was shown that the apparent viscosity decreased with shear rate, exhibiting the shear-thinning behavior of double emulsions. The observation that the decrease in viscosity was a function of the shear rate could be attributed to the structural breakdown of water droplets in emulsion and their rearrangement in the micro-structure, or the decrease in physical interactions between the phases [64]. Double emulsions are generally non-Newtonian fluids because of their complex rheological behaviors [65]. The double emulsions CX1, PX1, SX1 and WX1 had lower viscosities than CX2, PX2, SX2 and WX2, respectively, and therefore had more physical stability. Sample WX2 exhibited the highest viscosity and formed a thick layer around the droplets, increasing their size and, consequently, decreasing their stability. These results were also confirmed by the optical images and the wide variation observed in the droplet size of the WX2. A similar behavior was reported by Mohammadi et al. [66] for a W_1_/O/W_2_ stabilized with WPI; those authors described the thickening effect of biomolecules as the main factor in the stabilization of multiple emulsions. In comparison to WX1, SX1 and PX1 were more stable due to their lower viscosity values, as shown in Figure 4. Briefly, the viscosity of the W_1_/O/W_2_ also varied according to other factors, such as the creaming, nature, and behavior of the macromolecules in the continuous phase; the release of core substances into the external phase; and the extent of the interactions between droplets, the size of droplets, and the size distribution of droplets.

#### 3.2.3. Encapsulation Efficiency

**Figure 5:**
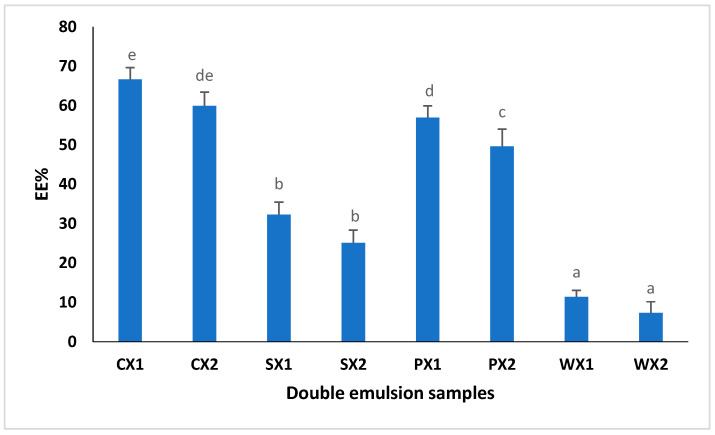
Encapsulation efficiency (EE%) of double emulsions emulsifying at two different times (C: Control, P: PPI, S: SPI, W: WPI; X1: 4065× *g*/1 min and X2: 4065× *g*/30 s; n = 3); Values followed by different letters are significantly different (*p* < 0.05).

EE% is defined as the percentage of bioactive components encapsulated during the internal water phase; thus, it is an indicator of the success of the various encapsulation methods [67,68]. In this study, the effect of various hydrophilic emulsifiers, such as proteins (SPI, PPI, and WPI), on EE% was studied (Figure 5). The use of Tween 80 as a food emulsifier is related to potential health risks; thus, it has been suggested that it should be replaced with food-grade natural emulsifiers. The EE% of the double emulsions varied between 7% and 66%. The results showed that the EE% of the W_1_/O/W_2_ prepared from SPI and PPI was significantly higher than that of the W_1_/O/W_2_ prepared using WPI (*p* < 0.05). Our findings indicated that the EE% was largely dependent on the type of emulsifier used in the continuous phase; thus, they were well aligned with the findings of Cuevas-Bernardino [67], who encapsulated quercitin in PPI, SPI and WPI gum complexes and reported that the quercitin-encapsulating efficiency was significantly better when using a PPI and SPI matrix. We postulated that plant proteins, specifically PPI, may offer higher affinity toward the MLE extract, thereby contributing to a higher EE%. Therefore, the utilization of plant proteins has great potential for encapsulating the phenolic compounds of an MLE compared with animal proteins.

## 4. Conclusions

This study showed that the predicted model obtained by RSM using a Box–Behnken design successfully evaluated the effect of independent variables (ethanol concentration, time, and amplitude) on the outcomes (TPC and antioxidant activity) of UE extraction of peppermint leaves. Additionally, this study demonstrates the feasibility of MLE encapsulation using emulsion-based systems, as it was effectively incorporated into stable W_1_/O/W_2_ emulsions. Our study showed that better physical stability (droplet size and viscosity) and higher encapsulation efficiency was found for emulsions prepared from plant proteins (PPI and SPI) than for those produced using animal proteins (WPI), and this finding was also supported by the optical images. Thus, it can be concluded that plant proteins can be used as a potential alternative to WPI for the preparation of double emulsions because of their high encapsulation efficiency. Moreover, the findings of this study may provide valuable insights towards the formulation of new functional food products from encapsulated MLE with enhanced phenolic content and antioxidant activity. In the future, hydrodynamic cavitation could be employed for phenolic extraction, as upscaling of ultrasonic-assisted extraction can be cumbersome and expensive.

## Figures and Tables

**Table 1:** Experimental plan with coded and uncoded levels of independent variable amplitude, time and ethanol concentration.

	Coded Levels			Uncoded Levels			Responses	
Experiment No.	X_a_	X_t_	X_e_	X_a_	X_t_	X_e_	TPC * (mg Gallic Acid Equivalent/g Mint Powder)	Inhibition (%)
1	−1	0	−1	20	20	70	49.94 ± 1.67	26.44 ± 0.95
2	0	−1	1	35	10	90	28.93 ± 2.54	17.26 ± 1.70
3	1	0	1	50	20	90	36.16 ± 2.9	27.45 ± 2.24
4	0	0	0	35	20	80	56.83 ± 1.38	31.68 ± 3.60
5	−1	−1	0	20	10	80	57.85 ± 3.05	16.88 ± 3.20
6	0	−1	−1	35	10	70	61.56 ± 3.05	45.77 ± 1.63
7	−1	0	1	20	20	90	31.23 ± 1.09	11.92 ± 3.67
8	−1	1	0	20	30	80	56.16 ± 4.14	27.21 ± 1.90
9	0	1	1	35	30	90	48.52 ± 2.01	21.63 ± 1.63
10	0	0	0	35	20	80	60.86 ± 2.29	32.14 ± 0.01
11	0	1	−1	35	30	70	66.34 ± 1.3	29.62 ± 2.31
12	0	0	0	35	20	80	61.02 ± 5.45	28.08 ± 0.03
13	1	1	0	50	30	80	58.63 ± 2.62	19.52 ± 2.31
14	1	−1	0	50	10	80	60.09 ± 5.45	35.19 ± 0.03
15	1	0	−1	50	20	70	58.15 ± 2.18	45.67 ± 4.90

* TPC- Total phenolic content, X_a_—Amplitude (%), X_t_—time (min), X_e_—ethanol concentration (%).

**Table 2:** Design summary and estimated regression coefficients for dependent variables and their significance.

	Constant	X_a_	X_t_	X_e_	X_a_ * X_a_	X_t_ * X_t_	X_e_ * X_e_	X_a_ * X_t_	X_a_ * X_e_	X_t_ * X_e_	R^2^ Value	Model *p*-Value
Total phenolic content	58.78 ***	NS	NS	−11.39 ***	NS	NS	−11.27 **	NS	NS	NS	93%	<0.05
% Inhibition	30.63	5.67 **	NS	−8.65 **	NS	NS	NS	−6.5 **	NS	5.13 *	86%	<0.1

NS—Non-significant terms; X_a_—Amplitude (%), X_t_—time (min), X_e_—ethanol concentration (%); *** *p* < 0.001, ** *p* < 0.05, * *p* < 0.1.

## Data Availability

Raw data will be provided on request.
